# Protection of CpG islands from DNA methylation is DNA-encoded and evolutionarily conserved

**DOI:** 10.1093/nar/gkw258

**Published:** 2016-04-15

**Authors:** Hannah K. Long, Hamish W. King, Roger K. Patient, Duncan T. Odom, Robert J. Klose

**Affiliations:** 1 Department of Biochemistry, University of Oxford, Oxford, OX1 3QU, UK; 2 Molecular Haematology Unit, Weatherall Institute of Molecular Medicine, University of Oxford, John Radcliffe Hospital, Oxford, OX3 9DS, UK; 3 Cancer Research UK Cambridge Institute, University of Cambridge, Cambridge, CB2 0RE, UK

## Abstract

DNA methylation is a repressive epigenetic modification that covers vertebrate genomes. Regions known as CpG islands (CGIs), which are refractory to DNA methylation, are often associated with gene promoters and play central roles in gene regulation. Yet how CGIs in their normal genomic context evade the DNA methylation machinery and whether these mechanisms are evolutionarily conserved remains enigmatic. To address these fundamental questions we exploited a transchromosomic animal model and genomic approaches to understand how the hypomethylated state is formed *in vivo* and to discover whether mechanisms governing CGI formation are evolutionarily conserved. Strikingly, insertion of a human chromosome into mouse revealed that promoter-associated CGIs are refractory to DNA methylation regardless of host species, demonstrating that DNA sequence plays a central role in specifying the hypomethylated state through evolutionarily conserved mechanisms. In contrast, elements distal to gene promoters exhibited more variable methylation between host species, uncovering a widespread dependence on nucleotide frequency and occupancy of DNA-binding transcription factors in shaping the DNA methylation landscape away from gene promoters. This was exemplified by young CpG rich lineage-restricted repeat sequences that evaded DNA methylation in the absence of co-evolved mechanisms targeting methylation to these sequences, and species specific DNA binding events that protected against DNA methylation in CpG poor regions. Finally, transplantation of mouse chromosomal fragments into the evolutionarily distant zebrafish uncovered the existence of a mechanistically conserved and DNA-encoded logic which shapes CGI formation across vertebrate species.

## INTRODUCTION

DNA methylation on CpG dinucleotides in vertebrate genomes is associated with transcriptional repression (1,2) and is epigenetically inherited during cell division to propagate repressive chromatin states (3–5). Conversely, short contiguous roughly 1–2 kb regions of CpG-rich DNA, known as CpG islands (CGIs), are interspersed throughout the genome and are resistant to DNA methylation (6,7). CGIs are found associated with 60–70% vertebrate gene promoters and are a central and evolutionarily conserved feature at these sites (8,9). CGIs function to recruit a family of ZF-CxxC DNA binding domain-containing proteins that associate with chromatin-modifying activities to remodel chromatin structure at gene promoters and contribute to gene regulation (10–12). Importantly, promoter-associated CGIs are usually free of DNA methylation regardless of the transcriptional state of the associated gene and are hypomethylated in most tissues (13–16), with only a subset of weak promoter-associated CGIs undergoing changes in DNA methylation during development (14,17–19). In addition to promoter-associated CGIs, an additional class of hypomethylated elements has been identified away from gene promoters that appear to function as distal gene regulatory elements, often encompassing enhancers (8,13,15,16,20). These regions tend to be hypomethylated in a subset of tissues, suggesting that the mechanisms underpinning their methylation state may differ from that of classical promoter-associated CGIs.

Despite our ever-increasing knowledge of DNA methylation landscapes in diverse vertebrate genomes, the principles and mechanisms that specify these patterns and protect CGIs and distal regulatory elements from DNA methylation is underexplored, particularly with respect to how these mechanisms function in their normal genomic context on chromosomes and during animal development. This is due to the fact that most of our information about these processes has relied on interrogating CGIs and their methylation states by inserting short DNA sequences (natural or synthetic) into engineered acceptor sites in cell culture systems (12,21–24). These studies have suggested that nucleotide features may function as a molecular signal to specify regions that should be refractory to the placement of DNA methylation (12,21–24). Alternatively, in some instances sequence-specific transcription factor binding has been linked to protection of the underlying DNA sequence from DNA methylation (22,23,25–27). Given that genomic context and tissue specific features play central roles in the way DNA elements function, it still remains poorly understood how significant these, or other mechanisms, are in specifying how hypomethylated regions of DNA (HMRs) form at a chromosome scale in animals.

To explore the mechanisms that shape CGI methylation state in a genomic context and to directly test whether these properties are evolutionarily conserved in vertebrates we have exploited a transchromosomic mouse animal model (Tc1) (28) in which most of human chromosome 21 has been stably transplanted into the mouse nuclear environment. Using genomic approaches we examined how HMRs are specified within 42 Mb of this human chromosome in developmentally distinct tissues. This revealed that irrespective of host species, CpG-rich promoter-associated CGIs are almost invariantly hypomethylated. In contrast, we discovered that distal elements are prone to alternative DNA methylation states depending on the host species and that this relies on both DNA sequence and transcription factor binding. Strikingly, these observations hold true when mouse chromosomal fragments are transposed into zebrafish, demonstrating for the first time that these general mechanisms are functionally conserved across divergent vertebrate species. Together this reveals that a DNA sequence-encoded logic and evolutionarily conserved mechanisms relying on the interplay between DNA features and transcription factor binding shape the DNA methylation based epigenome in vertebrate chromosomes.

## MATERIALS AND METHODS

### Tc1 mouse sample preparation

The Tc1 mouse line was maintained as previously described (28). Tc1 mice were bred by crossing female Tc1 mice to male (129S8 × C57BL/6J) F1 mice and were housed in the Biological Resources Unit under Home Office Licence (PPL 80/2197). Genomic DNA was extracted from fresh frozen Tc1 mouse liver and testis tissue using Genomic-tip 100/G kit (QIAGEN).

### Transposition of BAC DNA sequences into the zebrafish genome

Four mouse BACs were engineered to contain a GFP reporter driven by the eF1alpha promoter, and an inverted Tol2 transposition cassette (iTol2) flanking an ampicillin resistance gene (29) by BAC recombineering (Gene Bridges). BAC DNA was prepared using the Nucleobond BAC preparation kit and transposase RNA was generated using the SP6 RNA polymerase mMessage Machine kit (Ambion). BAC DNA and transposase RNA were introduced into zebrafish embryos by microinjection soon after fertilisation. GFP positive embryos were collected at 28–30 hpf and genomic DNA was extracted using the QIAGEN DNeasy Blood and Tissue Kit.

### BioCAP

BioCAP-sequencing was performed as described previously (30) in Tc1 mouse and Tc0 (wildtype) mouse testis and liver tissue in biological duplicate, and for zebrafish embryos containing integrated mouse BAC DNA. Human and mouse BioCAP datasets were detailed previously (GSE43512).

### HMR peak-calling and differential methylation analysis

Tc1 BioCAP-sequencing reads were aligned to a composite genome containing mouse chromosomes and human chromosome 21 using bowtie (31). HMRs were identified in Tc1 mouse tissues from BioCAP-sequencing traces using MACS1.4 (32) with settings –tsize = 50 –bw = 300 –mfold = 10,30 –pvalue = 1e-5 –verbose = 10 -g 4.8e+8 and including the use of an input control. Only HMRs that were identified in both biological replicates were retained. Human HMRs are detailed in (8). The human chromosome 21 in the Tc1 mouse has some rearrangements and duplications (33). To account for this, HMRs overlapping breakpoints or deleted regions were removed from the analysis and read values were scaled appropriately in duplicated regions. Differential methylation between species was identified if an HMR exhibited a greater than 2-fold change in BioCAP reads between the human and Tc1 mouse experiments using BAM files normalised to the same read count across chromosome 21. To compare HMRs on mouse chromosomes, HMR sites were identified in the Tc1 and Tc0 (wildtype) mouse using MACS1.4 (32) and compared as indicated above. Zebrafish Bio-CAP reads were aligned to a composite genome containing zebrafish chromosomes and each individual BAC sequence using bowtie-2 (34).

### Determining CpG density and GC content

CpG density and GC content of individual HMR regions was calculated based on the underlying DNA sequence for HMRs that were shared between human and Tc1 mouse or were species-specific. A matched background control was generated by randomly shifting HMR coordinates to another position on chromosome 21. CpG density and GC content was plotted as frequency distribution plots for liver and testis tissue.

### Genomic repeat age analysis

Repeat elements that overlapped the summit of HMR elements were analysed for repeat age. Repeat age was estimated by determining the number of substitutions from the repeat consensus sequence (Repeat-Masker track from UCSC, milliDiv column) and dividing this number by the estimated mutation rate for mammalian species, which is 2.2 × 10^−9^ per year (35,36). The distribution of repeat age was displayed as box plots for shared and species-specific HMRs.

### Transcription factor binding

Transcription factor binding sites were identified using the MACS1.4 peak caller with the same settings as were used to identify HMRs above (32). Shared or species-specific transcription factor binding events were determined by intersection of transcription factor binding sites identified in human and Tc1 mouse.

### Gene expression analysis

Gene expression data (reads per kilobase per million mapped reads, RPKM) was used to compare the expression of genes with an HMR overlapping their promoter (TSS ± 500bp) (36). Genes with a promoter-associated HMR were segregated depending on whether the HMR was shared between human and Tc1 mouse, or was species-specific.

### Graphical representation

Heatmaps and metaplots were generated using Homer annotatePeaks and plotted in R. Venn diagrams were plotted in R using the package VennDiagram. Boxplots were plotted in R using the package boxplot. Scatterplots were generated in R.

### Multiplex bisulfite sequencing

Bisulfite conversion of 250 ng human and two replicates of Tc1 mouse liver genomic DNA was performed using the EZ DNA Methylation-Gold Kit (Zymo Research). 10 picogram (pg) unmethylated and 10 pg methylated *Arabidopsis thaliana* BAC DNA (F24B22 and F19K16 respectively) was spiked into the three conversion reactions to control for bisulfite conversion (Diagenode DNA Methylation control package, EF-100-0040). PCR-amplified DNA was pooled for each replicate sample and Illumina sequencing libraries were prepared using the NEBNext DNA Library Prep Master Mix Set for Illumina (NEB). Indexed libraries were combined and sequenced on a MiSeq machine (Illumina). Sequencing data was analysed using the BisMark software from Babraham Bioinformatics (http://www.bioinformatics.babraham.ac.uk/projects/bismark/) (37). The two Tc1 biological replicates were highly similar, and were averaged for visualisation. Primer sets used for bisulfite sequencing are available on request.

## RESULTS

### Hypomethylated regions (HMRs) on human chromosome 21 are largely recapitulated in the transchromosomic mouse model

We recently demonstrated that many features of the CGI system are shared across vertebrate species (8).This led us to hypothesize that the mechanisms that specify DNA methylation state at CGIs may be evolutionarily conserved and rely on the underlying DNA sequence. To directly test this hypothesis, we exploited a transchromosomic mouse model (known as Tc1) that has been engineered to contain approximately 42 Mb of human chromosome 21 on a single independently segregating chromosome (28). This interesting trans-species model system provides a unique opportunity to ask how hypomethylated regions of DNA (HMRs) are formed and maintained in their natural genomic context in a developing animal following chromosome-scale introduction of DNA from one vertebrate, human, into the nuclear environment of second vertebrate, mouse. Importantly, it also allowed us to ask whether human chromosomal DNA-encoded information is sufficient to recapitulate the human host DNA methylation patterns in the nuclear environment of mouse, and in doing so test whether these mechanisms are evolutionarily conserved during development.

To answer these fundamental questions we isolated DNA from Tc1 mouse liver and testis and used biotinylated CxxC affinity purification (BioCAP) coupled to massively parallel sequencing to isolate regions of the genome containing hypomethylated DNA, and mapped these regions onto the mouse genome plus human chromosome 21 (30,38). We chose liver and testis to use as experimental tissues because their structure is highly similar in mammals in terms of cellular composition (39–41) and gene expression profiles (42–44), enabling a meaningful and direct comparison of BioCAP-seq signal on human chromosome 21. BioCAP experiments were carried out in duplicate with individual experiments correlating extremely well (*R*^2^ > 0.95, Supplementary Figure S1), further supporting the robustness of our approach (8,30). To ensure that the presence of the human chromosome did not affect the function of the DNA methylation system in the Tc1 mouse, we compared HMRs on the mouse chromosomes in the Tc1 mouse to HMRs formed in the same tissues in wild-type mice of the same genetic background. Importantly, HMRs on the Tc1 mouse chromosomes were indistinguishable from those of wild-type mice (Supplementary Figure S2A-F). Therefore, the presence of human chromosome 21 in the Tc1 mouse does not affect the function of the host DNA methylation machinery, indicating that the Tc1 mouse is a suitable model to study HMR formation on the newly introduced human chromosome 21.

**Figure 1.**
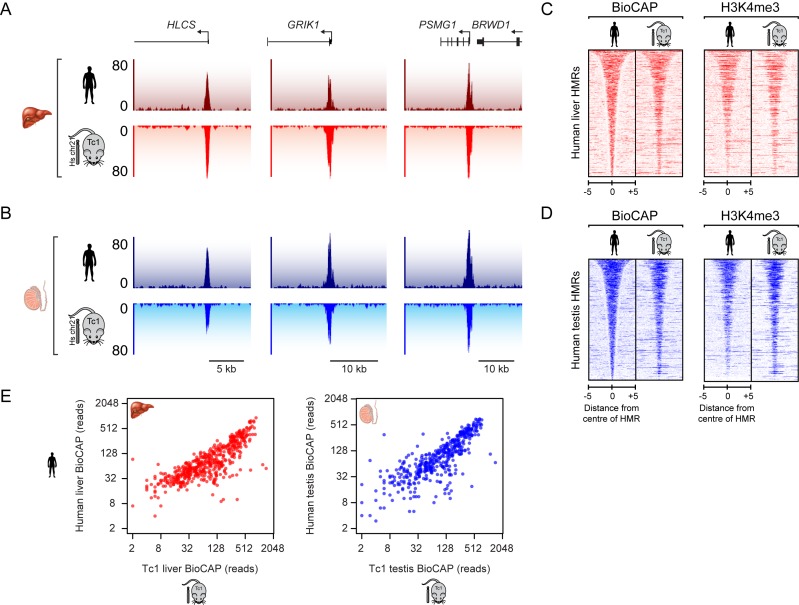
Hypomethylated regions (HMRs) on human chromosome 21 are largely recapitulated in the transchromosomic mouse model. (**A** and **B**) Profiles of non-methylated DNA (BioCAP-seq) at three regions on human chromosome 21 in human (upper) and in Tc1 mouse (lower, inverted) liver (A) and testis (B) tissues. Genes are depicted above the BioCAP traces. (**C** and **D**) Heatmaps depicting BioCAP (left) and H3K4me3 ChIP-seq (right) signal across human chromosome 21 HMRs in the human and Tc1 mouse liver (C) and testis (D) tissues. Signal is ranked according to HMR length and aligned to the centre of the HMR. Scalebar in kb. (**E**) Scatterplots of BioCAP-seq reads for human and Tc1 mouse at all human HMRs from liver (left) and testis tissue (right).

To investigate how HMRs form on human chromosome 21 in the mouse nuclear environment, we identified HMRs in developmentally distinct testis and liver tissue from the Tc1 mouse and directly compared these to HMRs formed on chromosome 21 in the corresponding human tissues (8). This revealed that the majority of human HMRs (85% in testis and 82% in liver) were recapitulated in the Tc1 mouse (Figure 1A and B). This was evident when BioCAP signal was plotted at all human chromosome 21 HMRs in the human and corresponding Tc1 mouse tissues (Figure 1C and D, left, and Figure 1E). To ensure that the HMRs formed on human chromosome 21 in the Tc1 mouse were functioning as they normally do in human tissues, we examined whether they were also modified by H3K4me3, a histone modification placed at HMRs by ZF-CxxC domain containing histone methyltransferase complexes (12,45). In agreement with the observation that the majority of HMRs form normally on human chromosome 21 in the Tc1 mouse tissues, we also observed robust H3K4me3 ChIP-seq signal at these sites in liver and testis tissue (Figure 1C and D, right). Overall, the majority of HMRs on human chromosome 21 and their stereotypical chromatin modifications are faithfully recapitulated when the chromosome is transplanted into mouse. This indicates that, for the most part, DNA sequence is sufficient to shape DNA methylation state at HMRs during vertebrate development, and furthermore that the systems required to achieve HMR formation at these sites are highly conserved between two vertebrate species, human and mouse.

### Identification of species-specific HMRs

**Figure 2.**
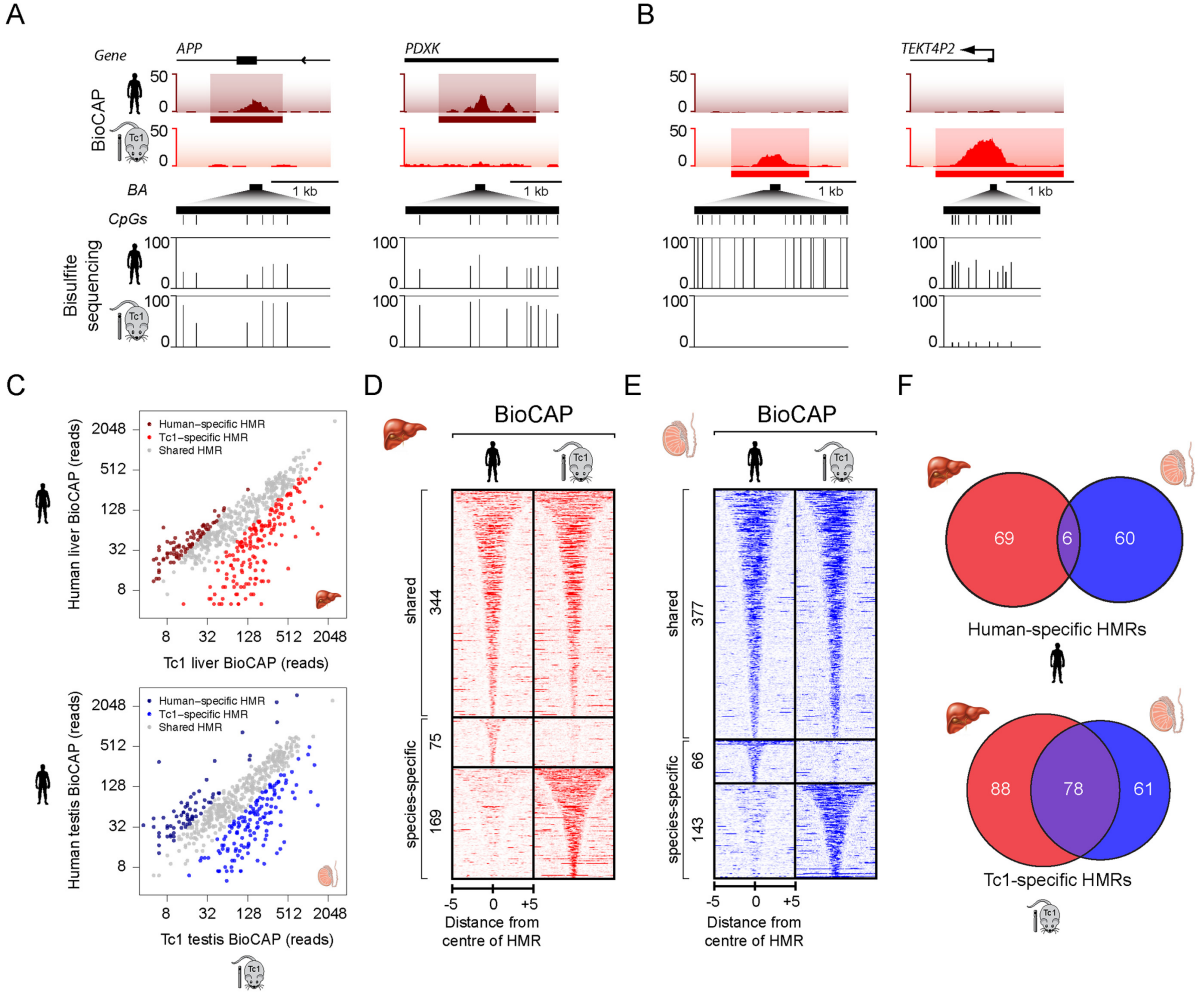
Identification of species-specific HMRs. (**A** and **B**) BioCAP-seq traces across two human-specific (A) and two Tc1-specific (B) HMRs on human chromosome 21. Species-specific HMRs (ssHMRs) are indicated by a horizontal bar below the BioCAP-seq traces (upper). Bisulfite sequencing at species-specific HMRs confirms alterations in methylation at these sites (lower). Bisulfite amplicons (BA) are depicted by a horizontal black bar, CpG dinucleotides by a vertical line and the methylation status of each CpG in human or Tc1 liver is depicted as a vertical line between 0 and 100%. (**C**) Scatter plot of BioCAP-seq reads for human and Tc1 mouse at all HMRs to illustrate human and Tc1-specific HMRs in liver (upper) and testis (lower). (**D** and **E**) Heatmaps of BioCAP-seq signal in human and Tc1 mouse liver (D) and testis (E) tissues illustrate that a subset of HMRs are differentially methylated. Heatmaps are ranked according to HMR length and aligned to the centre of the HMR with shared (upper), human-specific (middle), and Tc1-specific (lower) sites clustered together. Scalebar in kb. (**F**) Venn diagrams depicting the overlap between human-specific HMRs (upper) or Tc1-specific HMRs (lower) from different tissues.

Although HMRs on chromosome 21 were for the most part appropriately specified in the Tc1 mouse, we observed a number of locations where DNA methylation was gained or lost in the Tc1 mouse when compared to the same locus in human (Figures 1E and 2A and B, upper panels). This interesting observation suggests that some species-specific features must also contribute to HMR specification. To explore these changes in more detail, we used targeted deep bisulfite sequencing to examine at single base pair resolution the DNA methylation state at a number of these loci. In all cases, this revealed quantitative differences in DNA methylation across a range of CpG densities in agreement with the altered magnitude of BioCAP-seq signal. This is evident when individual loci were visualized (Figure 2A and B, lower panels) and more broadly at a set of 37 differentially methylated HMRs but not control loci (Supplementary Figure S3). We refer to these differentially methylated loci as species-specific HMRs (ssHMRs) to distinguish them from the majority of HMRs that display invariant methylation patterns when chromosome 21 resides in either Tc1 mouse or human tissues.

Differential analysis of BioCAP-seq signal over the combined set of Tc1 and human HMRs revealed a surprisingly large number of ssHMRs (244 in liver and 209 in testis) (Figure 2C–E). At these sites there was a tendency for the hypomethylated state to predominate in the Tc1 tissue, accounting for 68–69% of ssHMRs (Figure 2C–E). Interestingly, human-specific HMRs were usually unique to one tissue (96%) (Figure 2F, upper), suggesting that HMR formation at these sites may rely on tissue-specific events. In contrast, fewer Tc1-specific HMRs were unique to one tissue (66%) (Figure 2F, lower) suggesting that species-specific, but tissue-invariant, activities contribute to HMR formation at the remaining (33%) Tc1-specific HMRs.

### The majority of species-specific HMRs are not directly related to changes in gene expression and are distal to gene promoter regions

**Figure 3.**
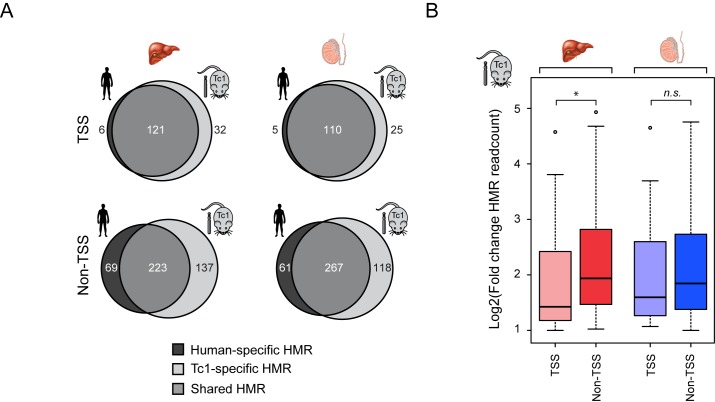
The majority of species-specific HMRs are distal to gene promoter regions. (**A**) Venn diagrams depicting the overlap between HMRs in the human and Tc1 mouse tissue. HMRs are segregated into those located at transcription start site (TSS ± 500bp) (upper) and those found away from TSSs (lower). Human-specific HMRs are coloured in dark grey, shared HMRs in grey and Tc1-specific HMRs in light grey. (**B**) Boxplots depicting fold-change in BioCAP signal between Tc1 mouse and human at Tc1-specific HMRs located at a gene TSS or elsewhere in the chromosome. Tc1-specific HMRs away from gene TSSs exhibit a greater fold-change in BioCAP signal compared to those at gene TSSs. Significance values calculated using Welch's *T*-test (*P < 0.05).

The identification of ssHMRs on human chromosome 21 in the human and mouse nuclear environments provided a unique opportunity to examine these differences in detail and discover the mechanisms that specify these methylation patterns. Given the historical relationship between CGIs and gene promoters, we first examined whether ssHMRs were associated with gene transcriptional start sites (TSSs). This revealed that ssHMRs are infrequently associated with TSSs (38/244 for liver and 30/209 for testis), and that the majority of these sites are located away from gene promoters (Figure 3A). Furthermore, when we examined the magnitude of change in BioCAP signal at ssHMRs, those found away from gene promoters showed larger alterations suggesting that these sites are more prone to nuclear environment-dependent changes in DNA methylation (Figure 3B). In the small number of instances where ssHMRs overlapped a gene promoter, there was no obvious relationship between the observed differences in DNA methylation and host gene expression patterns, suggesting that these alterations are not related to transcriptional changes (Supplementary Figure S4).

### Species-specific HMRs in the Tc1 mouse are CpG-rich and often associated with young repetitive DNA elements

**Figure 4.**
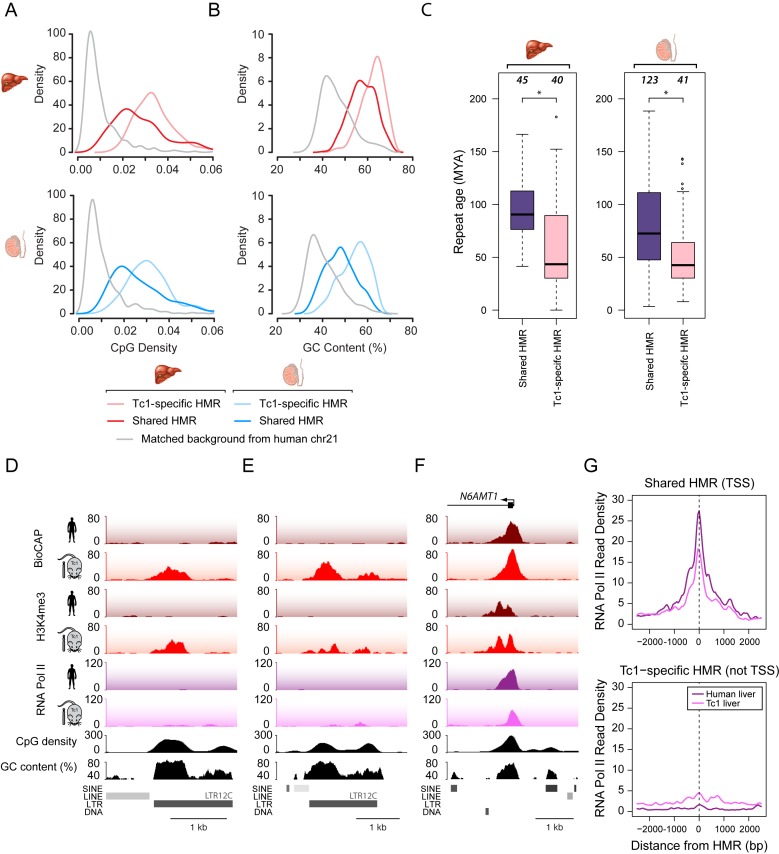
Tc1-specific HMRs are CpG-rich and often associated with young repetitive DNA elements. (**A** and **B**) CpG density (A) and GC content (B) plots of liver (upper panel, red) and testis (lower panel, blue) HMRs that are shared between human and Tc1 mouse (dark line) or are Tc1-specific (pale line). A background control is indicated (grey). (**C**) Boxplots depicting age of repeats (in million years) associated with HMRs in liver (left) and testis tissue (right). HMRs are segregated into those that are shared in human and Tc1 mouse (purple) and are Tc1-specific (pink). The difference in repeat age between shared and Tc1-specific HMRs is significantly different as calculated by a Mann–Whitney U test (*P < 5 × 10^−4^). (**D** and **E**) Snapshots of two Tc1-specific HMRs associated with a repetitive element. (**F**) A snapshot of a gene promoter on human chromosome 21 with a shared HMR, and which is associated with RNA PolII in both the Tc1 mouse and endogenous human nuclear environment. (**G**) RNA PolII enrichment at human-Tc1 shared TSS-associated HMRs (upper) and Tc1-specific non-TSS-associated HMRs (lower) in human and Tc1 mouse liver.

Given the overrepresentation of ssHMRs in regions of the genome distal to TSSs, we examined in more detail the DNA sequence features at these sites to understand if this could explain their species-specific methylation patterns and provide insight into the mechanisms shaping their methylation state. We initially focused on CpG dinucleotide frequency and GC content, based on our previous observations that these nucleotide features are almost universally elevated amongst regions in vertebrate genomes that lack CpG methylation (8). Initially we examined Tc1-specific HMRs as they were the most abundant group of ssHMRs and directly compared their nucleotide features to shared HMRs. In general, shared HMRs on human chromosome 21 in both the human and Tc1 mouse were characterized by classical CpG island nucleotide features, with CpG density and GC content above the genome average, consistent with their invariantly hypomethylated state (Figure 4A and B). Somewhat surprisingly, Tc1-specific HMRs had highly elevated CpG density and GC content, even eclipsing the nucleotide frequencies characteristic of the non-methylated state at most shared HMRs (Figure 4A and B). This indicates that despite their CGI-like nucleotide composition, a subset of CpG and GC-rich regions on human chromosome 21 are actively targeted by the DNA methylation machinery in human tissues, and yet these same sequences evade DNA methylation in the Tc1 mouse.

CpG and GC-rich sequences that are methylated in human tissues are often associated with repetitive or parasitic DNA elements (46). Targeted methylation of these DNA elements is a common evolutionary strategy to suppress the potentially deleterious effects on the host genome (1,47). In the human genome, primate-specific repetitive elements have emerged recently enough that their CpG frequency remains high in the face of evolutionary mechanisms that drive down the overall frequency of CpG dinucleotides in vertebrate genomes (48–52). Therefore some of these elements display an elevated CpG density compared to the surrounding genome, much like CGI elements (51,53). We previously showed that in some cases, primate-specific repeats can possess latent gene regulatory potential and become activated in the mouse (36). When the average age of repeats associated with either shared HMRs, human-specific HMRs, and Tc1 mouse-specific HMRs were compared it was evident that young, newly acquired repetitive elements were overrepresented in Tc1 mouse-specific HMRs (Figure 4C and Supplementary Figure S5). Interestingly, however, loss of methylation at most of these sites was unrelated to gene transcription, as examination of ChIP-seq for RNA polymerase II (Pol II) in liver tissue at Tc1-specific HMRs revealed that only a subset (20%) of these sites acquired RNA Pol II occupancy. This is evident at individual loci (compare Figure 4D and E to F) and through analysis of RNAPII occupancy at Tc1-specific HMRs compared to transcribed shared HMRs (Figure 4G). Together, this indicates that Tc1-specific HMRs do not simply result from unmasking of dormant or cryptic gene promoters with latent regulatory potential (36) and supports the idea that species-specific trans-acting factors actively target DNA methylation to these sites. Therefore, our observations reveal that in the absence of co-evolved mechanisms that specifically methylate young human repeat elements, elevated CpG and GC content is sufficient to protect these sequences from DNA methylation during development. Together these interesting interspecies observations provide new and widespread evidence that mechanisms that sense CpG dinucleotide frequency contribute centrally to evasion of the DNA methylation machinery.

### Species-specific transcription factor binding is widely associated with species-specific HMR formation

**Figure 5.**
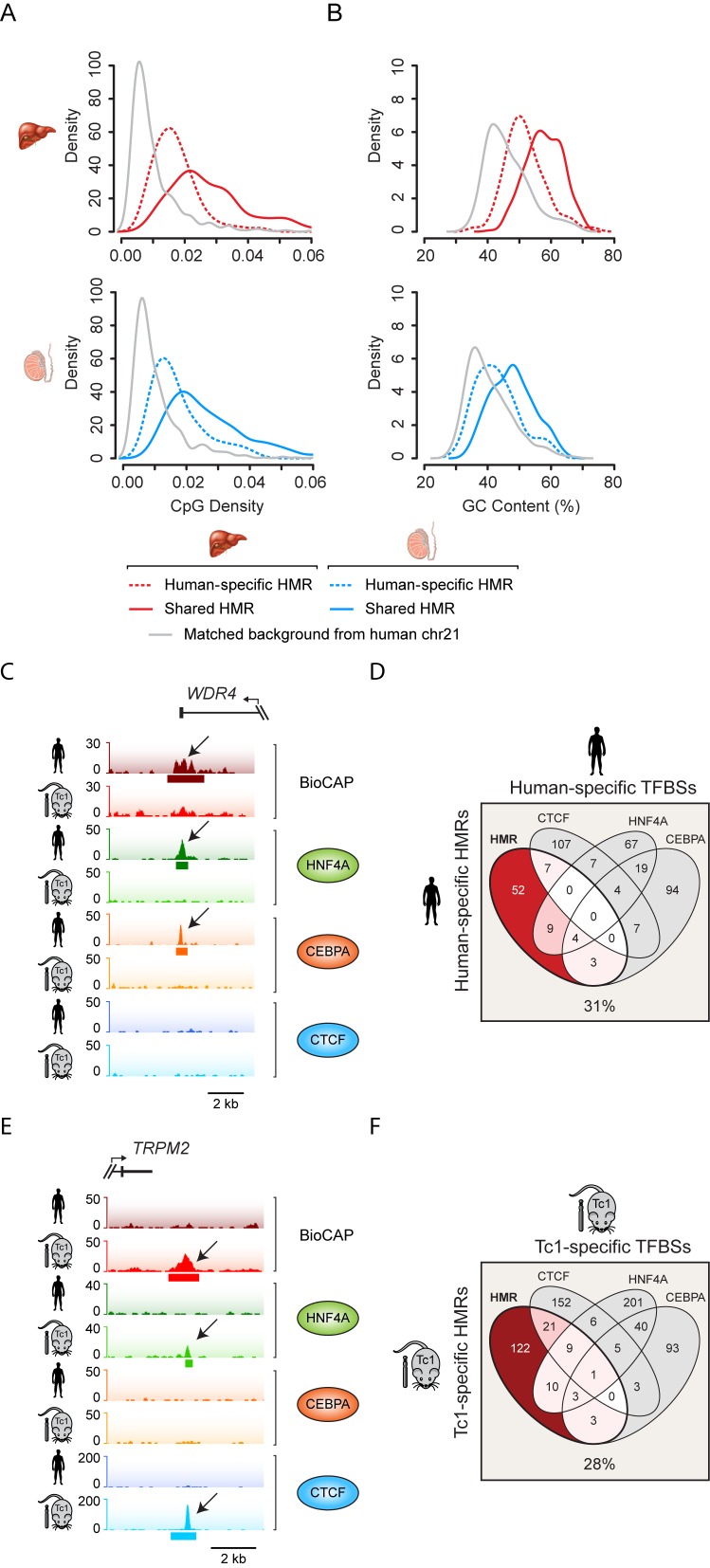
Species-specific transcription factor binding is widely associated with species-specific HMR formation. (**A** and **B**) CpG density (A) and GC content (B) plots of liver (upper panel, red) and testis (lower panel, blue) HMRs that are shared between human and Tc1 mouse (dark line) or are human-specific (dashed line). A background control is indicated (grey). (**C**) A snapshot illustrating a human-specific HMR corresponding to a human-specific transcription factor binding event as indicated by CEBPA and HNF4A ChIP-seq signal. (**D**) A 4-way Venn diagram comparing human-specific HMRs to human-specific transcription factor binding sites (TFBSs) for CEBPA, HNF4A and CTCF on human chromosome 21. 31% of human-specific HMRs overlapped a human-specific TFBS. (**E**) A snapshot illustrating a Tc1 mouse-specific HMR corresponding to a Tc1 mouse-specific transcription factor binding event as indicated by HNF4A and CTCF ChIP-seq signal. (F) A 4-way Venn diagram comparing Tc1-specific HMRs to Tc1-specific transcription factor binding sites for CEBPA, HNF4A and CTCF on human chromosome 21. 28% of Tc1-specific HMRs overlapped a Tc1-specific TFBS.

Elevated nucleotide features at young human repetitive sequences appear to contribute to their hypomethylated state in the Tc1 mouse, however this cannot explain how a subset of human HMRs on human chromosome 21 acquire DNA methylation in the mouse nuclear environment. To understand the features that drive formation of these human-specific HMRs we again examined their underlying nucleotide features. In contrast to Tc1 mouse-specific HMRs, human-specific HMRs were characterized by CpG density and GC content above the genome average, but significantly below those found at classical CGI elements (Figure 5A–B). This suggests that mechanisms protecting these sites from methylation in human tissues do not rely on classical CGI-like nucleotide frequencies.

We and others have recently identified a subset of HMRs that are differentially methylated depending on tissue type (8,14–17,54). These tissue-specific HMRs often correspond to distal regulatory elements, including enhancers. Much like the human-specific chromosome 21 HMRs, these sites have low CpG dinucleotide frequency and GC content. Distal regulatory elements usually correspond to sites of transcription factor occupancy, which has been proposed to protect the underlying binding site and surrounding DNA from methylation (20,22,23,26,27,55). Therefore, an interesting possibility is that human-specific HMRs at regions exhibiting an intermediate-to-low CpG frequency and GC content may result from species-specific DNA binding events. To investigate this possibility, we examined the ChIP-seq profiles of three DNA binding factors CEBPA, HNF4A and CTCF in the human and Tc1 mouse liver (36). Despite the conserved nature of these DNA binding factors between human and mouse, we identified a large number of species-specific DNA binding events on chromosome 21, likely due to subtle changes in binding site preference (Supplementary Figure S6A). An examination of these species-specific transcription factor binding events revealed that human-specific HMRs frequently overlap transcription factor binding events that are unique to the human liver. For example, the 3′ end of the *WDR4* gene is characterized by HNF4A and CEBPA binding events that only occur in the human liver, and this corresponds to a human-specific HMR (Figure 5C). Strikingly, 31% (23/75) of human-specific HMRs intersected at least one transcription factor binding event that was unique to the human liver tissue (Figure 5D and Supplementary Figure S6B). Therefore, differential transcription factor binding appears to be a central feature of species-specific methylation states at sites with intermediate CpG and GC content on human chromosome 21.

Given the clear overlap between transcription factor binding and ssHMRs in human tissue, we wondered whether Tc1 mouse-specific transcription factor binding events may also be implicated in the methylation state of some ssHMRs in mouse. When we examined Tc1-specific HMRs we again observed that nearly a third (47/169) of these sites overlapped with a Tc1-unique transcription factor binding event (Figure 5F). For example, downstream of the human *TRPM2* gene an HMR is hypomethylated in the Tc1 mouse and bound by both HNF4A and CTCF. In human these binding events are absent and the region is methylated (Figure 5E and Supplementary Figure S6C). Strikingly, the methylation state of approximately one-third of all species-specific chromosome 21 HMRs appear to be related to the binding profiles of just three transcription factors (Figure 5D and F). This suggests that if more transcription factors were examined, the methylation state of a large proportion of species-specific chromosome 21 HMRs might correspond to these events. Therefore together, our *in vivo* interspecies experiments demonstrate the breadth with which DNA binding transcription factor occupancy can shape DNA methylation profiles at sites in the genome that have low CpG density and GC content, an observation that is further supported by recent *in vitro* experiments that also suggest DNA binding events contribute to local methylation profiles (20,22,23).

### The principles shaping HMR formation are DNA encoded and conserved across vast expanses of divergent vertebrate evolution

Although it is clear in the Tc1 mouse that a subset of sites on human chromosome 21 can exist in alternative methylation states depending on the host species, the majority of HMRs throughout the human chromosome form completely normally (Figure 2C–E). This suggests that the general mechanisms specifying DNA methylation patterns and protecting CGIs from methylation are for the most part intact and conserved across 75 million years of evolutionary divergence between human and mouse. We recently demonstrated that features of a CGI-like system exist in most, if not all, branches of vertebrate evolution (8). If DNA sequence is indeed the central driver in HMR formation and the mechanisms driving these methylation states are functionally conserved in divergent vertebrate species then one would predict that the transplantation of mammalian DNA sequences into even more distantly related vertebrates would also result in the accurate specification of HMRs during animal development.

**Figure 6.**
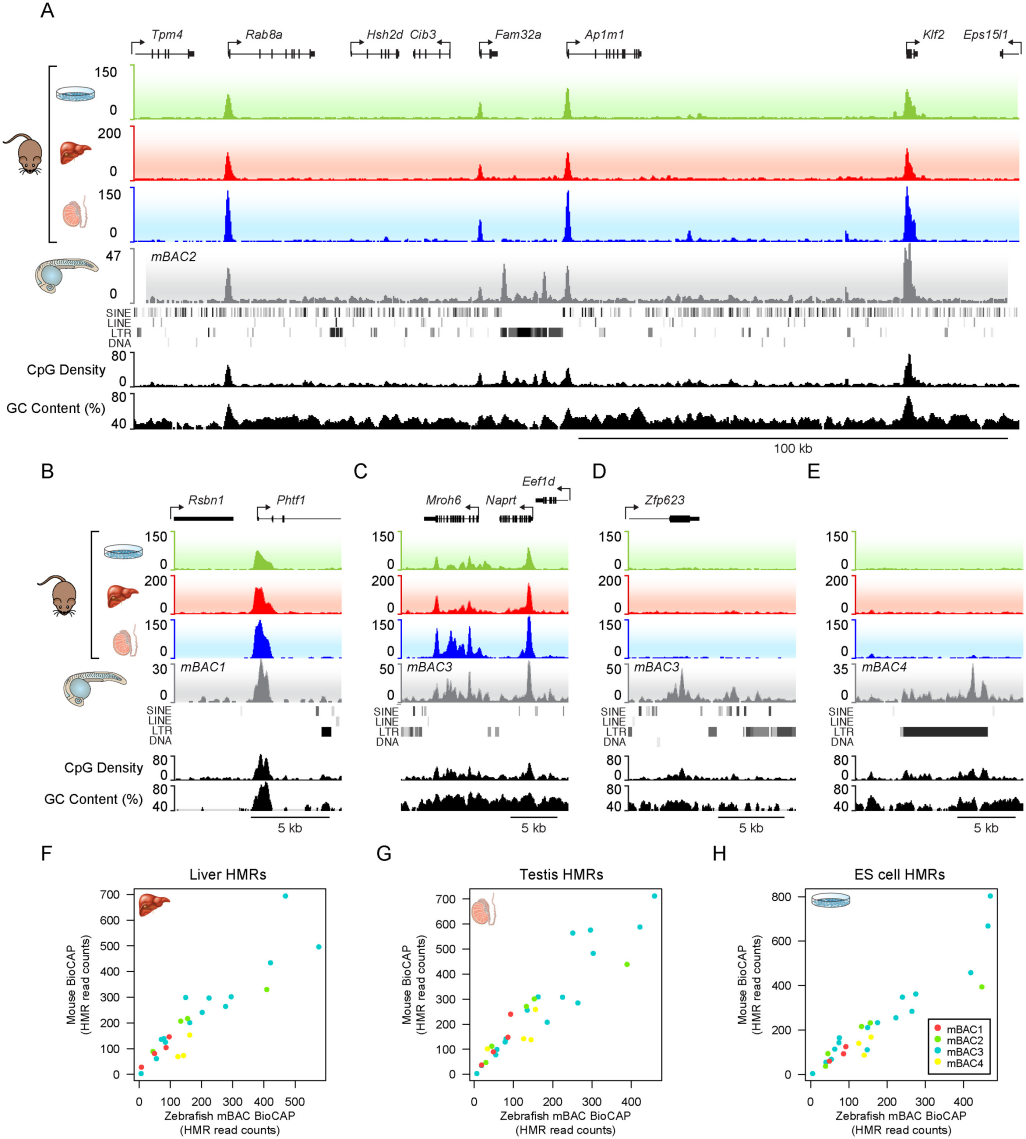
The DNA encoded principles that underpin HMR formation are conserved across vast expanses of divergent evolution. (**A**) BioCAP-seq profile of a mouse chromosomal DNA fragment introduced into the zebrafish genome and analysed at 28–30 h post-fertilisation (hpf). The BioCAP signal from three representative mouse cell-types ES cells, liver and testis (green, red and blue traces) and the BioCAP trace observed for this locus in the developing zebrafish (grey) is indicated. CpG density and GC content are depicted in black. All four mouse HMRs form on the mouse BAC DNA in the zebrafish embryo and a cluster of repetitive LTR elements in the centre of the mouse BAC form zebrafish-specific HMRs. (**B**) A snapshot of a promoter-associated mouse HMR. (**C**) A snapshot of a broadly hypomethylated region. (**D**) A snapshot of a zebrafish-specific HMR region that forms at a CpG and GC-rich mouse exonic region that is normally methylated in mouse tissues. (**E**) A snapshot illustrating a cluster of mouse LTR elements which are CpG dense and become hypomethylated in zebrafish. (**F**–**H**) Scatterplots comparing BioCAP-seq read counts at mouse HMRs in zebrafish with mouse liver (F), testis (G) and embryonic stem cells (H).

To test this hypothesis, we introduced large chromosomal fragments of mouse DNA into the zebrafish genome and used BioCAP-seq to examine how the resulting methylation profiles compared to the profiles observed in mouse tissues. To achieve this, four mouse bacterial artificial chromosomes (mBACs) each containing approximately 200 kb of genomic sequence were introduced into the genome of the fertilized zebrafish zygote by Tol2-mediated transposition (29). When we examined the resulting methylation profiles of the mouse DNA sequences in 28–30 hours post fertilisation (hpf) zebrafish embryos, we observed that promoter-associated mouse HMRs were appropriately specified, including both typical 1–2 kb HMRs (Figure 6A and B) and broad HMRs, which tend to be associated with genes important for development (Figure 6C) and that the BioCAP-seq signal at HMRs on the mouse DNA in zebrafish correlated linearly with the BioCAP-seq signal in mouse tissues (Figure 6F–H). Together this indicates that the principles driving protection of these elements from DNA methylation are DNA encoded and conserved across vertebrate evolution.

Interestingly, a small number of new HMRs were formed on the mouse BACs integrated into zebrafish, much like the species-specific HMRs that were observed in the Tc1 mouse. When we examined these sites in more detail they again corresponded to CpG and GC rich regions within the mBAC including an exonic region of the Zfp623 gene (Figure 6D). Importantly, several sites where zebrafish specific HMRs formed in the mBACs corresponded to small clusters of CpG rich LTR repeats, including intracisternal A particles (IAPs) and the ERV1 element MMVL30-int, that are restricted to the mouse lineage, and are absent from the zebrafish genome (Figure 6A and E, Supplementary Figure S7A). Many of the LTR retrotransposons present on the mBACs exhibit CpG density comparable to endogenous CpG islands (Supplementary Figure S7B) and, therefore, in a manner similar to young primate specific repeat sequences in the Tc1 mouse, zebrafish presumably lacks co-evolved mechanisms to drive DNA methylation to these mouse repeat regions. Consequently, the elevated CpG and GC content of these repeat sequences appears to render them refractory to methylation further supporting the argument that nucleotide content is a central and evolutionarily conserved DNA encoded feature shaping HMR formation.

Therefore, by examining the principles that shape HMR formation *in vivo* across distinct vertebrate organisms that have undergone extensive divergent evolution, we find that nucleotide content, in particular CpG and GC richness, is a central feature shaping HMR formation. Furthermore, the fact that most mouse HMRs, when situated in their normal genomic context, formed accurately in zebrafish (Figure 6F–H) reveals quite remarkably that the mechanisms driving stereotypical HMR formation, particularly at gene promoters, are highly conserved, despite 450 million years divergent evolution.

## DISCUSSION

The mechanisms that form and maintain epigenomes *in vivo* are very poorly understood. Recently, cell culture-based approaches inserting small DNA fragments into the genome have suggested some principles that may contribute to formation of DNA methylation states (22–24). However, these approaches do not examine how DNAs behave in their natural genomic context, are limited in the total amount of DNA sequence they interrogate, and do not capture the divergent developmental trajectories that ultimately shape chromosomal DNA methylation patterns in tissues. Therefore, to overcome these limitations we have exploited a transchromosomic mouse model and examined developmentally distinct tissues to discover that the majority of HMRs on human chromosome 21 are appropriately recapitulated when transplanted into the mouse nuclear environment, indicating that the underlying DNA sequence and genomic context is largely sufficient to drive the observed methylation patterns in vertebrate organisms.

A subset of regions distal to classical promoter-associated CGIs showed species-specific methylation patterns. Interestingly, CpG and GC-rich regions associated with young repeat sequences became hypomethylated in the mouse nuclear environment, and also in experiments where mouse BACs were integrated into zebrafish, apparently in the absence of mechanisms that would normally drive methylation to these sites. This feature is uniquely revealed through our interspecies experiments and demonstrates that the mechanisms which evolved to recognize repeat elements and methylate them can override the inherent capacity of CpG and GC-rich sequences to evade DNA methylation, presumably as a requirement to protect the genome against the activity of these potentially deleterious elements. These protection mechanisms may rely on the activity of piwi RNA (piRNA) clusters that encode a historical memory of transposition events from invading retroviruses and recruit PIWI proteins to endogenous transposons resulting in their methylation (56,57). Alternatively, a class of KRAB zinc finger DNA binding proteins appears to rapidly evolve the capacity to recognize emerging classes of repetitive DNA sequence and similarly targets them for DNA methylation (58,59). Importantly, in the absence of co-evolved mechanisms that target DNA methylation to these repetitive sequences, it appears that CpG and GC-richness renders these DNA sequences refractory to DNA methylation, much like classical HMRs, supporting the idea that the cell uses CpG and GC richness as a cue to protect against DNA methylation. In contrast to CpG and GC-rich regions, the methylation state of DNA sequences with intermediate CpG and GC content are frequently defined by species-specific DNA binding events, providing new widespread experimental evidence that site-specific DNA binding factor occupancy, in addition to DNA sequence features, can play a central role in shaping DNA methylation patterns at these sites. Together these observations reveal that CpG and GC nucleotide features and protein-based DNA binding events defined by DNA sequence are central determinants in shaping methylation patterns on chromosomal DNA sequences *in vivo* during development. Furthermore, by transplantation of mouse chromosome fragments into zebrafish we discovered that these general principles are conserved across vast expanses of divergent vertebrate evolution, uncovering the existence of a highly conserved and DNA encoded logic that shapes methylation patterns in the vertebrate epigenome.

Our observation that CpG and GC nucleotide content is a central determinant in protecting chromosomal DNA from DNA methylation is in agreement with observations that short synthetic or bacterial CpG and GC-rich DNA sequences can often evade the DNA methylation machinery when inserted into vertebrate genomes (12,21,22). This can in many instances be achieved even if sequences apparently lack motifs necessary for site-specific occupancy of DNA binding transcription factors, suggesting that alternative mechanisms must exist to sense and protect these CpG and GC-rich regions from DNA methylation. This could be achieved by evolutionarily conserved ZF-CxxC domain containing proteins that bind specifically to non-methylated CpG dinucleotides (10,60) and associate with chromatin modifying activities that create chromatin environments that are refractory to DNA methylation. For example, H3K4me3 is targeted to CGIs by several ZF-CxxC proteins (10,12) and can inhibit DNA methyltransferase function on chromatin *in vivo* (61–63). Furthermore, the KDM2B ZF-CxxC domain-containing protein functions as an H3K36me1/me2 demethylase and is part of the polycomb repressive complex 1 that appears to protect a subset of CGIs from DNA methylation (11,64–68). This occlusion of the DNA methylation machinery is likely reinforced by mechanisms that can actively remove DNA methylation, such as the TET oxygenases that are putative DNA demethylase enzymes and occupy CGIs via their ZF-CxxC domains (69–72). Together these observations suggest that chromatin-modifying activities targeted to CGIs by ZF-CxxC domain containing proteins may play a central and evolutionarily conserved role in protecting CGIs from DNA methylation and shaping DNA methylation landscapes. Furthermore, our demonstration that the mechanisms underpinning specification of HMRs are mechanistically conserved in zebrafish also suggests this tractable developmental model system could be exploited through morpholino or CRISPR based approaches to remove ZF-CxxC proteins or other contributing mechanisms, individually or in combination, to further dissect the mechanisms that shape HMR formation during development.

By examining methylation states across vast expanses of the same DNA sequence in two completely distinct vertebrate nuclear environments we provide extensive new evidence that transcription factor binding events are central determinants in shaping DNA methylation profiles at intermediate CpG and GC content regions of the genome. This parallels observations that dynamic and differential methylation is often observed at CpG and GC-poor enhancer elements across tissues of individual organisms (8,14–17,54), a feature that in some cases has been attributed to DNA binding transcription factors occupancy (55,73,74). Importantly, our new trans-species observations provide chromosome-scale experimental evidence that transcription factor occupancy can help to shape DNA methylation patterns. This is supportive of other single gene studies that suggested that SP1 transcription factor occupancy could contribute to the DNA methylation state of the APRT1 gene (26,27), more limited mutational analysis of transcription factor binding sites (20,22,23), and the suggestion that single nucleotide variation associated with human disease affects the capacity of nuclear factors to read the underlying DNA sequence and potentially contributes to alterations in the epigenome (75,76). An important step moving forward will be to determine mechanistically how DNA binding factors influence DNA methylation. It appears unlikely that this will simply result from transcription factors protecting the underlying DNA from the methylation machinery, as HNF4A lacks CpG dinucleotides in its recognition sequence yet influences the methylation of surrounding CpG dinucleotides (73). One interesting possibility is that transcription factors could exploit active mechanisms, perhaps through the function of the TET DNA demethylases, to protect distal gene regulatory sites from the transcriptionally repressive influences of DNA methylation. In support of this possibility a number of recent studies have provided preliminary evidence that this may indeed be the case (55,77).

In summary, through exploiting transchromosomic animal experiments we discover that DNA methylation patterns *in vivo* are primarily informed and shaped by DNA sequence. In doing so we demonstrate that classical CGI like sequences in large genomic regions of DNA can be interpreted by evolutionarily conserved mechanisms, even in distantly related vertebrate organisms, to protect these sequences from DNA methylation and to shape the epigenome during development.

## ACCESSION NUMBERS

Datasets have been deposited in the Gene Expression Omnibus (GEO) with the accession number GSE72208.

## Supplementary Material

SUPPLEMENTARY DATA
