# Supplementary material for: Protection of CpG islands from DNA methylation is DNA-encoded and evolutionarily conserved
Source: Nucleic Acids Res. 2016 Apr 15;44(14):6693–706. doi: 10.1093/nar/gkw258 (PMC5001583; doi:10.1093/nar/gkw258)
Supplement: SUPPLEMENTARY DATA [file supp_44_14_6693__index.html]

Protection of CpG islands from DNA methylation is DNA-encoded and evolutionarily conserved — Protection of CpG islands from DNA methylation is DNA-encoded and evolutionarily conserved — SUPPLEMENTARY DATA 

# Protection of CpG islands from DNA methylation is DNA-encoded and evolutionarily conserved

## SUPPLEMENTARY DATA

- SUPPLEMENTARY DATA
