# Supplementary material for: Protection of CpG islands from DNA methylation is DNA-encoded and evolutionarily conserved
Source: Nucleic Acids Res. 2016 Apr 15;44(14):6693–706. doi: 10.1093/nar/gkw258 (PMC5001583; doi:10.1093/nar/gkw258)
Supplement: SUPPLEMENTARY DATA [file supp_gkw258_nar-03355-x-2015-File008.docx]

**
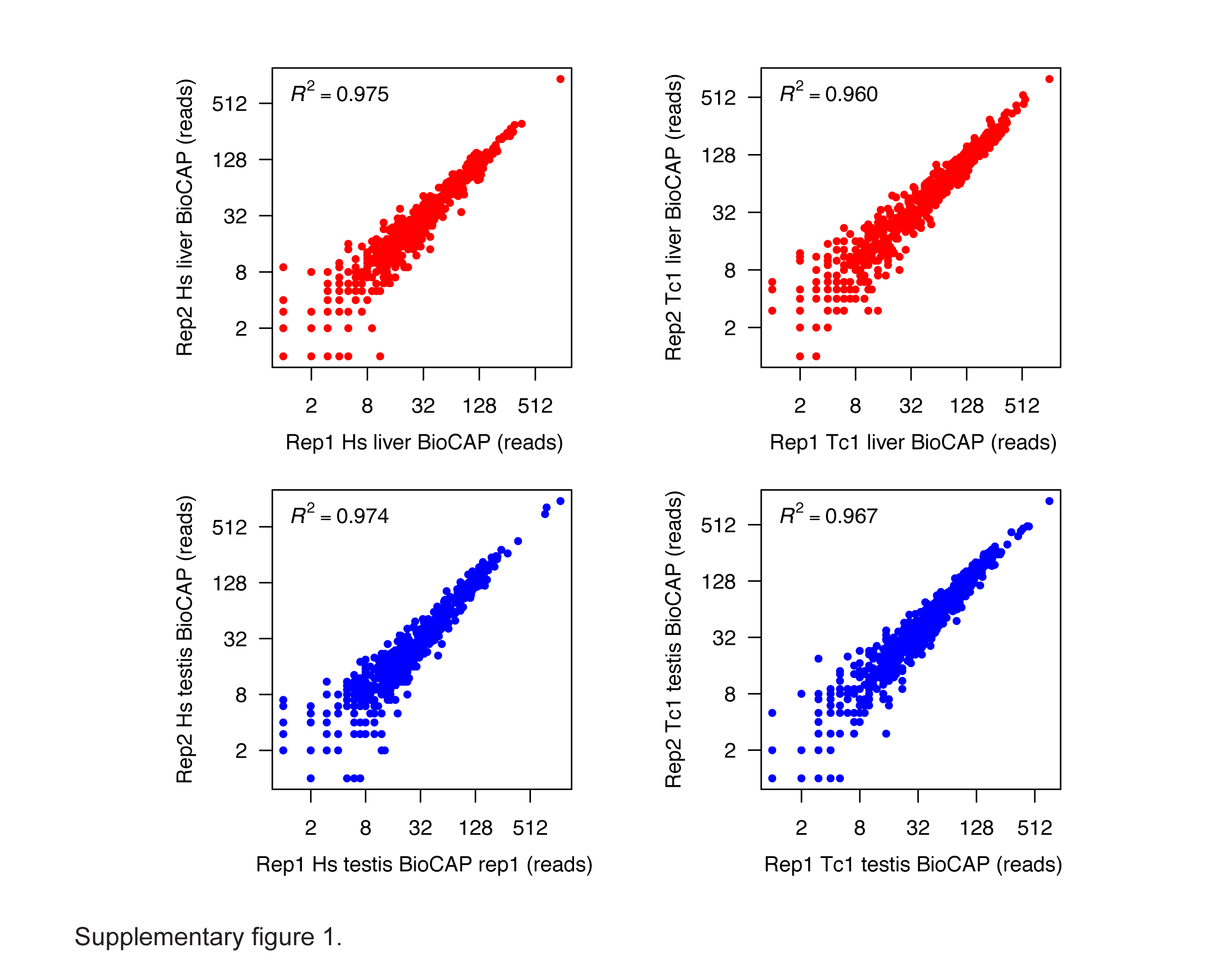
**

**Supplementary Figure 1: Replicate BioCAP datasets are highly correlated**

Replicate BioCAP experiments are highly correlated. Scatterplots depicting BioCAP-seq read counts for replicate BioCAP experiments at HMRs on chromosome 21 for human liver, human testis, Tc1 liver and Tc1 testis tissue. The correlation coefficient (R^2^ value) was greater than 0.95 in all cases.

**
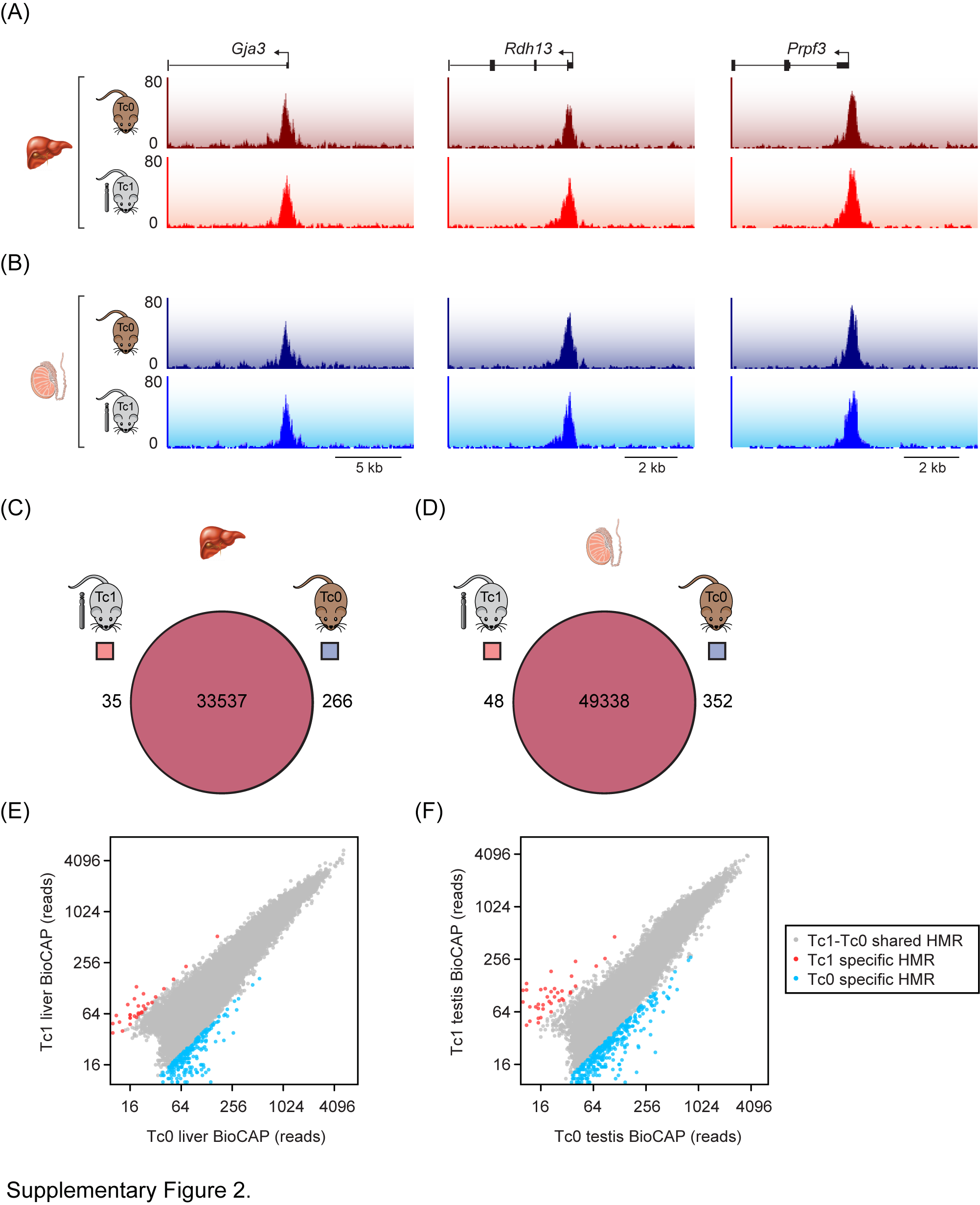
**

**Supplementary Figure 2: Mouse HMRs form normally in the Tc1 mouse**

(A-B) Three representative snapshots of HMRs on mouse chromosomes in the Tc1 mouse (lower) or matched Tc0 wildtype mouse (upper) in liver (A) or testis (B) tissue.

(C-D) Venn diagrams depicting the overlap of HMRs identified on mouse chromosomes in the Tc1 and Tc0 wildtype mouse in liver (C) and testis tissue (D).

(E-F) Scatterplots of BioCAP-seq read counts for HMRs identified on mouse chromosomes in the Tc1 and Tc0 wildtype mouse in liver (E) and testis tissue (F).


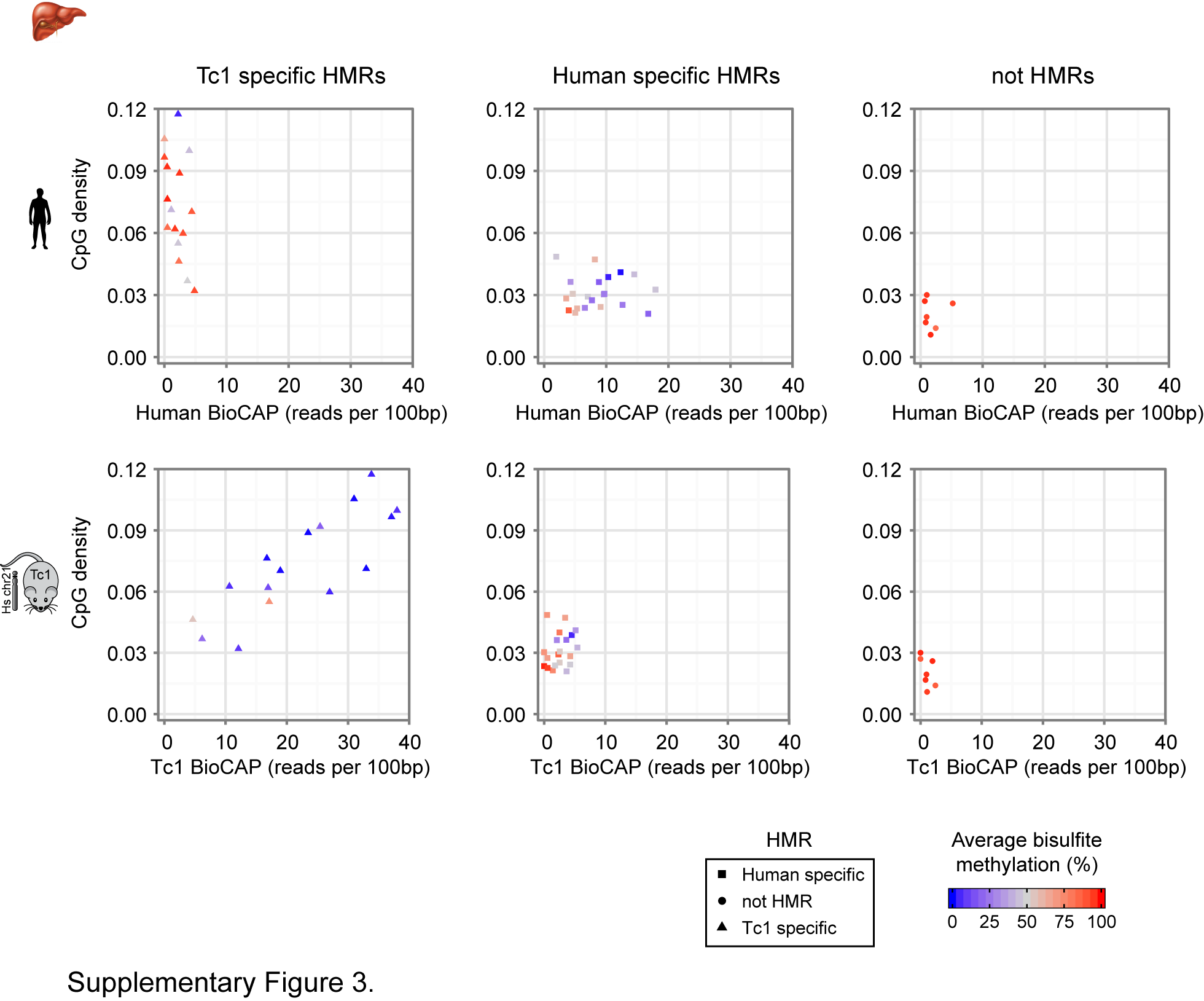


**Supplementary Figure 3: Multiplexed deep bisulfite sequencing based validation of species-specific HMRs**

Scatterplots comparing BioCAP-seq read counts to CpG density at 16 Tc1-specific HMRs (left), 21 human-specific HMRs (middle) and 7 random control regions (right). Deep sodium bisulfite sequencing analysis of DNA methylation is overlaid on these scatterplots (with methylation levels indicated by blue 0% to red 100% methylated) and validates that species-specific HMRs are accurately identified by BioCAP-seq across a range of CpG densities.

**
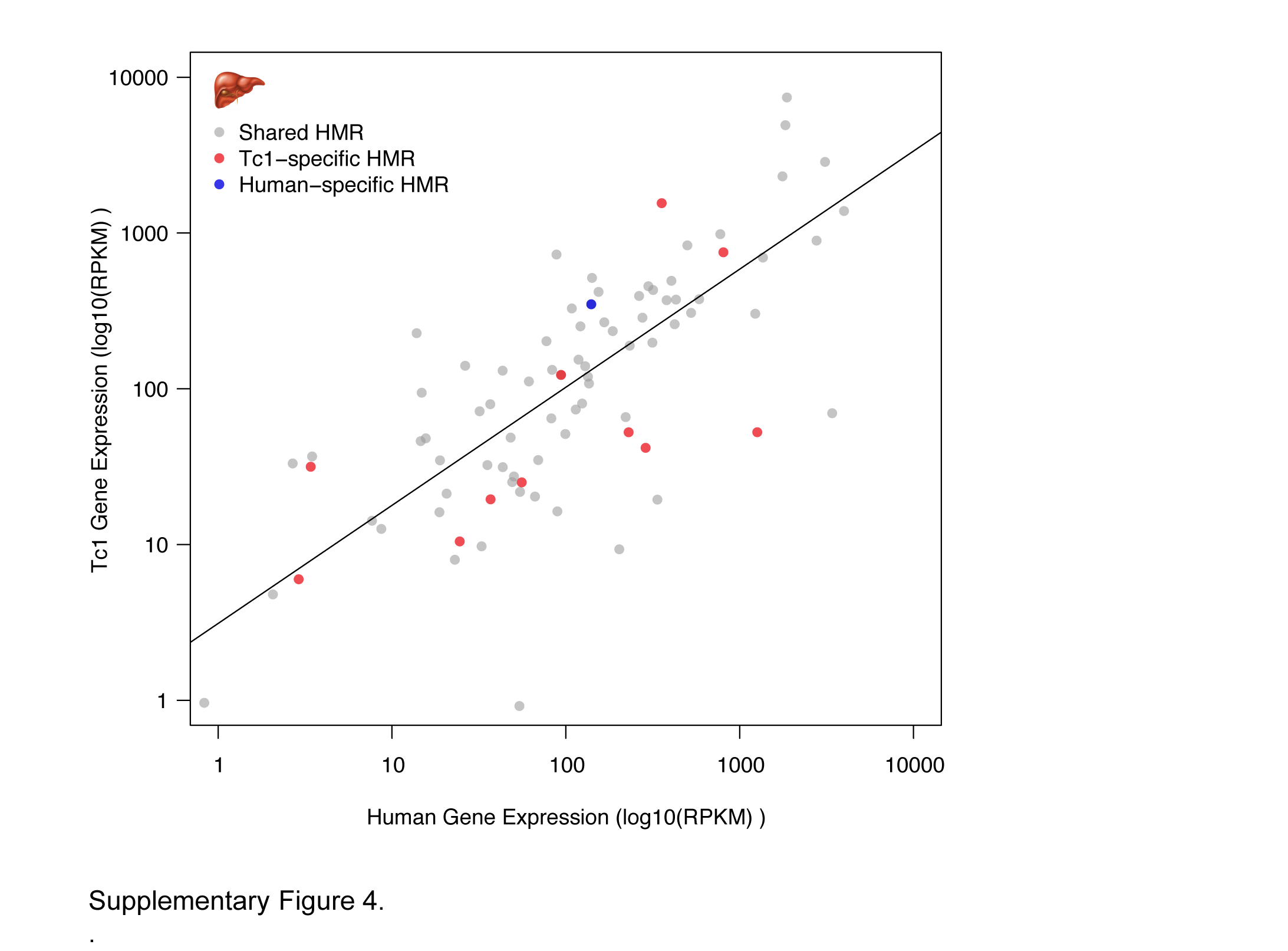
**

**Supplementary Figure 4: Comparison of gene expression levels at genes associated with increased promoter hypomethylation in the human or Tc1 mouse liver**

A scatter plot depicting gene expression levels for genes on chromosome 21 in the Tc1 mouse or human liver, plotted on a log10 scale. Genes with a species-specific HMR overlapping the TSS are highlighted in red (Tc1) or blue (human) and genes with a shared HMR overlapping the TSS are plotted in grey. Only genes which exhibited sufficient sequence coverage ([1](#_ENREF_1)) for differential gene expression analysis are plotted.


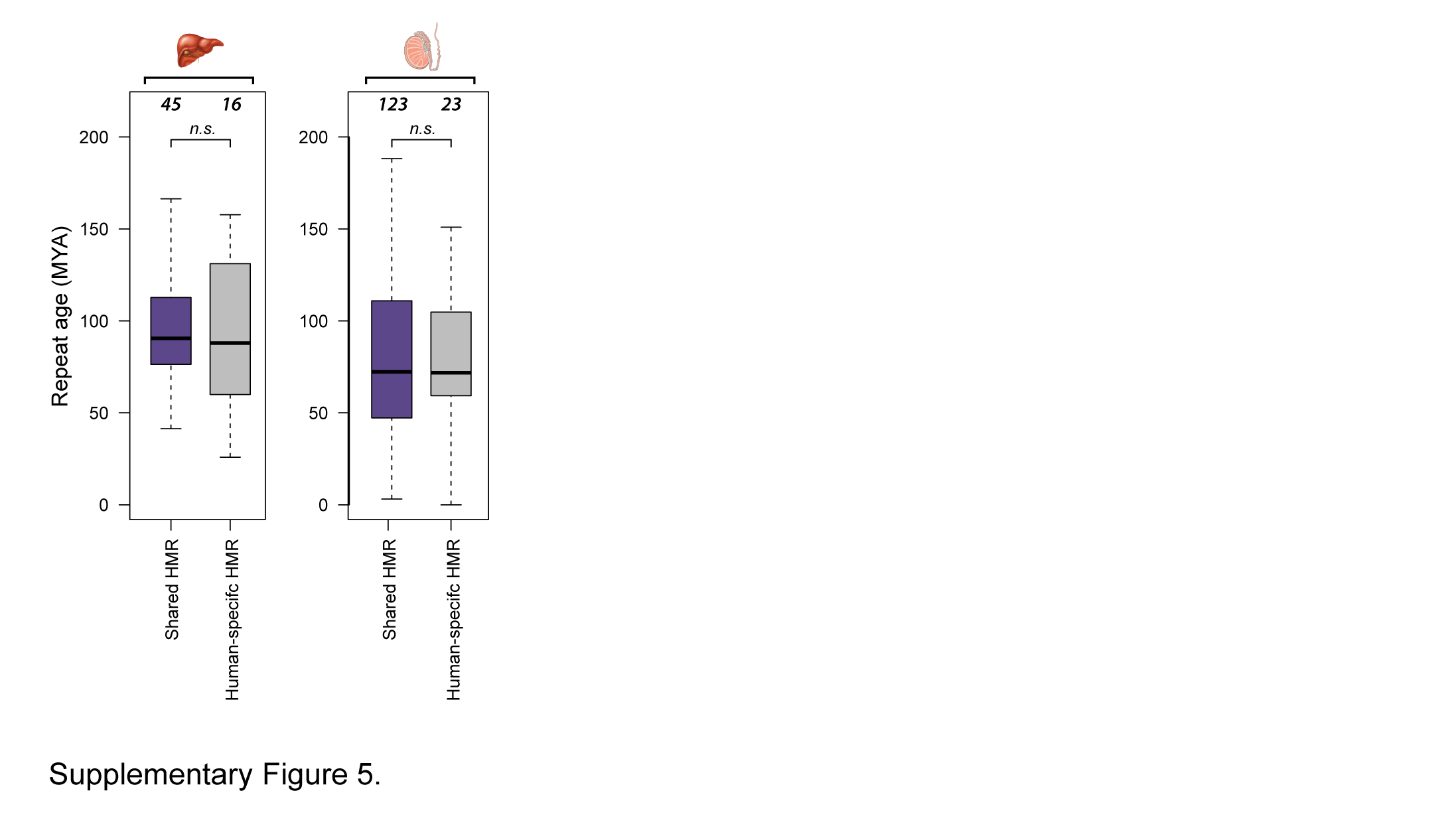


Supplementary Figure 5: Human-specific HMRs are not associated with young repetitive DNA elements

Boxplots depicting age of repeats (in million years) associated with HMRs in liver (left) and testis tissue (right). HMRs are segregated into those that are shared in human and Tc1 mouse (purple) and those that are human-specific (grey). The difference in repeat age between shared and human-specific HMRs is not significantly different (n.s.) as calculated by a Mann Whitney U test (p > 0.05).

**
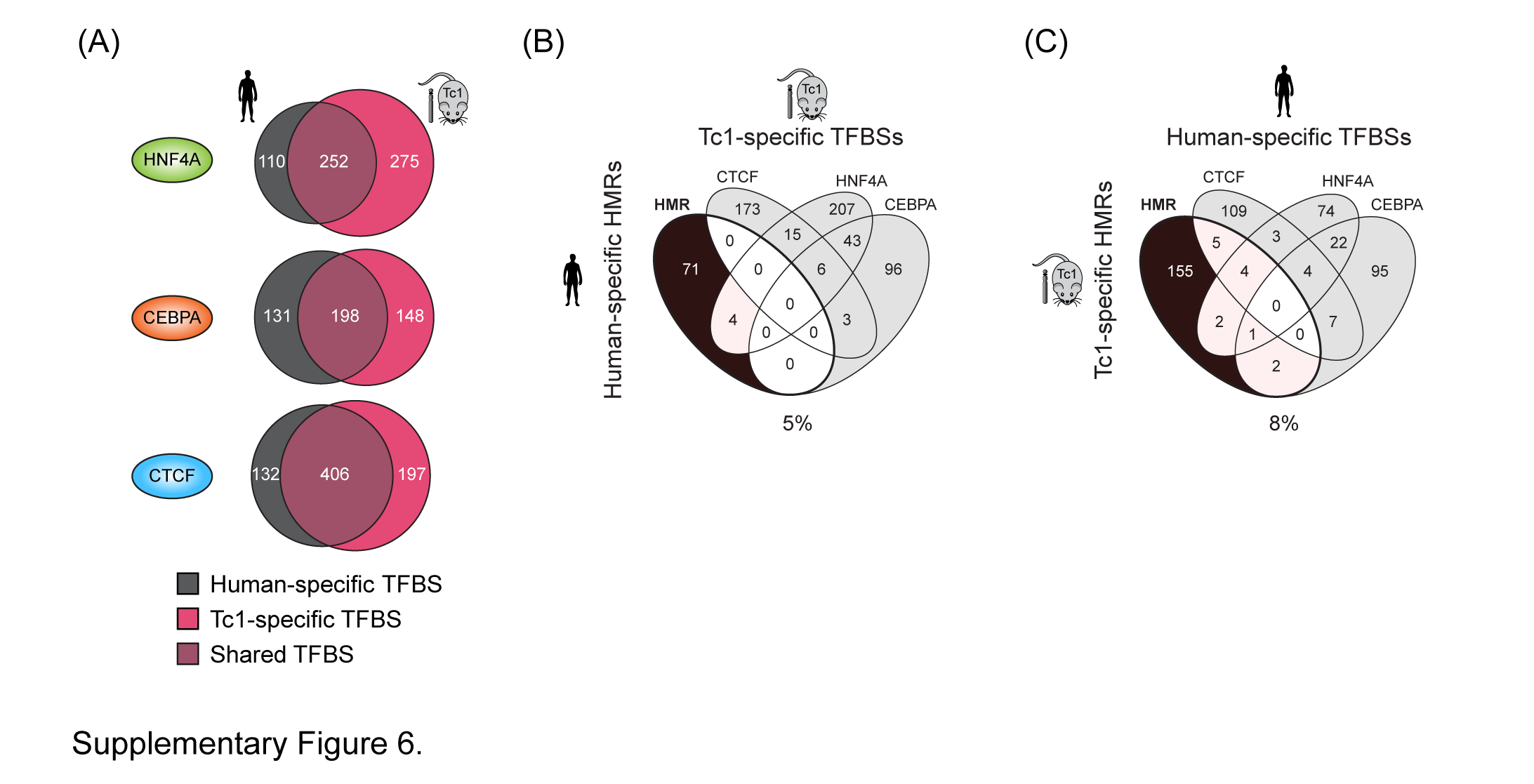
**

**Supplementary Figure 6: Shared and species-specific transcription factor binding on human chromosome 21**

(A) Comparison of transcription factor binding events on human chromosome 21 in human and Tc1 mouse liver for HNF4A, CEBPA and CTCF.
(B) A 4-way Venn diagram comparing human-specific HMRs to Tc1-specific transcription factor binding sites for CEBPA, HNF4A and CTCF on human chromosome 21. Only 5% of human-specific HMRs overlapped a Tc1-specific TFBS.

(C) A 4-way Venn diagram comparing Tc1-specific HMRs to human-specific transcription factor binding sites for CEBPA, HNF4A and CTCF on human chromosome 21. Only 8% of Tc1-specific HMRs overlapped a human-specific TFBS.


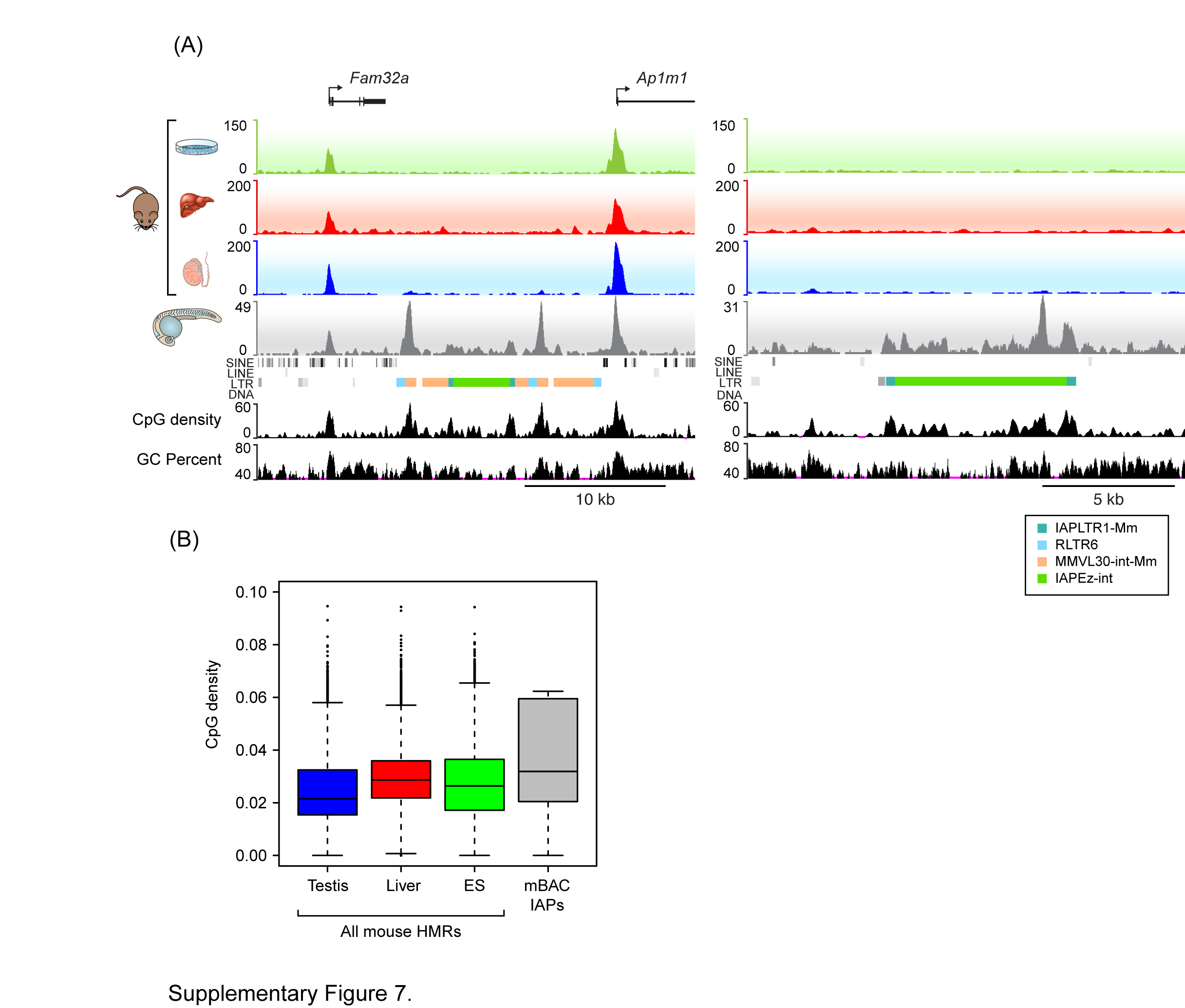


**Supplementary Figure 7: A subset of mouse-specific LTR retrotransposons resemble mouse CpG islands and form species-specific HMRs in zebrafish.**

(A) Snapshots of the two clusters of mouse-specific LTR retrotransposons on mouse BACs 2 (left) and 4 (right) that include IAP and MMVL30 LTRs. These are CpG rich and are hypomethylated in zebrafish.

(B) Boxplots depicting the distribution of CpG density at all HMRs from mouse testis (blue), liver (red) and ES cells (green) compared to IAP elements on mBAC2 and mBAC4 (grey).
